# Feeling Gratitude and Depletion: The Ambivalent Consequences of Receiving Help in the Workplace

**DOI:** 10.3390/ijerph18042039

**Published:** 2021-02-19

**Authors:** Yuanfang Zhan, Jinfan Zhou, Huan Cheng, Renyan Mu

**Affiliations:** 1 School of Management, Huazhong University of Science and Technology, Wuhan 430074, China; yuanfangzhan@hust.edu.cn (Y.Z.); chenghuan@whut.edu.cn (H.C.); 2 Department of Human Resource Management, School of Business, Nanjing University, Nanjing 210093, China; zhoujf@smail.nju.edu.cn; 3 School of Management, Wuhan University of Technology, Wuhan 430074, China

**Keywords:** helping behavior, organizational citizenship behavior, deviance, gratitude, ego depletion

## Abstract

Drawing from social exchange theory, we developed a dual-path model of employees’ reactions to episodic help received from colleagues. Through a diary study, using data collected from 127 full-time employees working in a large Chinese bank, we tested this model, revealing that receiving episodic help from colleagues is positively related to the help receivers’ gratitude and ego depletion. Through these two ambivalent psychological states, help receivers were found to simultaneously engage in more organizational citizenship behaviors and deviance behaviors on a daily basis. These empirical findings contribute to research that adopts a target-centric perspective in examining the consequences of helping behavior in the workplace.

## 1. Introduction

Interpersonal helping is affiliative, individual-focused organizational citizenship behavior (OCB) that refers to “individual behavior that is discretionary, not directly or explicitly recognized by the formal reward system, and in the aggregate promotes the efficient and effective functioning of the organization” [1]. It pervasively exists and is encouraged in organizations because lending help to others who have difficulty in fulfilling work requirements and tasks can benefit the organization and members inside the organization [2]. From the recipient’s point of view, previous studies suggested that receiving help was a double-edged sword. On the one hand, receiving help from coworkers instills in recipients a sense of felt obligation and gratitude for others’ goodwill [3,4] because receiving help is beneficial for them to recover from work-related hindrances and pressure [5]. On the other hand, receiving others’ help is likely considered as a threat to the recipient’s self-esteem, indicating inferiority, inadequacy, and dependency as a human being [6,7], and elicits recipient indebtedness [8], which might deplete the resources of recipients. Those recipients who feel grateful are, thus, more likely to reciprocate prosocially, such as engaging in OCBs, in the workplace [9], but receiving help also leads to negative emotions and a negative evaluation of the helper [10].

This positive reciprocal process of interpersonal help among employees within an organization demonstrates the norm of reciprocity as explained in social exchange theory [4]. Social exchange theory posits that reciprocity is the central norm held by two parties maintaining a social exchange relationship that is long-term-oriented [11]. Influenced by the norm of reciprocity, both parties believe that, in the long run, the mutual exchange of resources in their relationship will balance out [12]. Despite this positive reciprocal process in the long run, the immediate psychological reactions of help receivers after receiving episodic help and before they repay for the help remain underexamined. Exploring employees’ state reactions to episodic help received from colleagues is important because it is quite usual that employees may not be able to or cannot find a chance to repay those help givers immediately in an organizational context, because of the asymmetry of resources, status, and personal abilities between help giver and help receiver.

Interestingly, a handful of social-psychology studies revealed that individuals may react less positively toward received help or people lending help than what is expected [10,13,14]. Moreover, individuals may even generate negative emotions and pressure if they expect a low possibility to reciprocate [15,16]. Thus, it is theoretically meaningful to extend these social-psychology research findings to the organizational context and to examine how employees react to colleagues’ interpersonal help in the short run. Indeed, abundant empirical studies showed that employees’ state reactions have a significant impact on their short-term behaviors in the workplace [17,18].

In this research, we draw from social exchange theory [4,12] and develop a dual-path model of help receivers’ state reactions toward episodic help received in the workplace. Specifically, the reciprocity norm in social exchange suggests that individuals both appreciate and feel obliged to repay the kindness of others [8]. Focusing on this ambivalent psychological state, we examined employees’ two different psychological reactions to colleagues’ help at work. One is a state of gratitude, a moral emotion referring to “a feeling of appreciation in response to an experience that is beneficial to, but not attributable to, the self” [3]. The other is ego depletion, which refers to the depletion of one’s self-regulatory resources [19]. On the basis of these two psychological reactions, we further examine help receivers’ ambivalent behavioral outcomes in the workplace on a daily basis. Their grateful feelings could lead to the positive behavior of OCB, whereas their ego depletion leads to the negative behavior of deviance—intentional acts that violate the organizational norms or rules and cause actual harm to the organization or members inside the organization [20].

**Figure 1:**
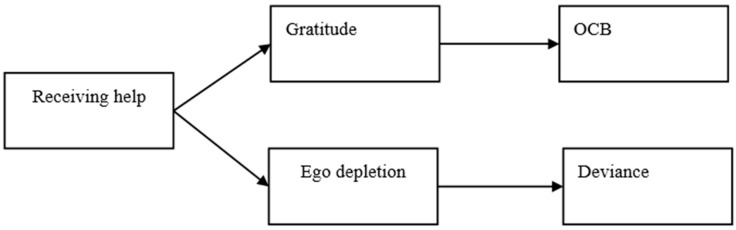
Overall conceptual model. OCB: organizational citizenship behavior.

Our paper makes two main contributions to the literature. First, we advance prior research of interpersonal help in the management literature by focusing on employees’ state reactions to their recipients of colleagues’ episodic help. Through a diary study, our findings reveal that, on a daily basis, help receivers feel both grateful and depleted by exerting self-control resources to regulate their inner desires to repay others’ kindness [21]. Second, we contribute to the OCB literature by enriching the energizing stream of research on OCB’s negative influences [22,23]. Specifically, an increasing number of studies reveal the negative effects of performing OCB on the actors’ psychological and behavioral outcomes at work [24]. Our findings revealed the other side of the coin by showing that individual-focused OCB (i.e., helping behavior) may also generate negative effects on targets’ psychological (i.e., ego depletion) and behavioral (i.e., deviance) outcomes in the short run. Our conceptual model is shown in Figure 1.

## 2. Theory Framework and Hypotheses

### 2.1. Gratitude as a Positive Reaction to Receiving Help

Gratitude is an emotion, an affective state that persists for a short period of time [25]. Recent emotion scholars regarded gratitude as a moral emotion that is associated with “the interests or welfare wither of society as a whole or at least of persons other than the judge or agent” [26]. Because gratitude reflects an individual’s appreciation to the experience that is not attributable to but can benefit themselves [3], it is a positive moral emotion and other-focused. Thus, experiencing others’ kindness that is beneficial to oneself can activate one’s positive feelings of gratitude. In the organizational context, employees are likely to generate feelings of gratitude when receiving help from others (leaders and coworkers) at work.

Interpersonal help is individual-focused OCB. Different forms of interpersonal help exist in organizations, such as instrumental helping, which refers to the problem-focused provision of interpersonal aid that aims at helping others in completing work goals and tasks [27], and emotional helping, which refers to the provision of sympathy, caring, empathy, understanding, and other emotion-related aids that aim at helping others recover from negative affective states [28]. Lending help to others, independently of the type of help, is beyond the employees’ in-role obligations, and doing so may not lead to obtaining formal rewards from the organization [1]. Thus, research revealed that helping behavior is resource-consuming to help givers [29]. On the other hand, employees feel gratitude when receiving help from colleagues who sacrifice personal resources (e.g., money, free time, and social relationships) to aid their work and/or affective problems in the workplace [3].

**Hypothesis** **1** **(H1).** *Receiving help is positively related to the recipient’s gratitude.*

### 2.2. Ego Depletion as a Negative Reaction to Receiving Help

In addition to generating the positive reaction of gratitude to interpersonal help, help receivers may also generate some negative reactions [6]. Several theoretical perspectives can account for recipients’ negative reactions to help. For instance, threat to self-esteem theory suggests that receiving help undermines the recipient’s competence feelings and perceived self-esteem [7]. In addition, reactance theory [30] suggests that people value freedom, and thus, an individual generates negative reactions to others’ help, especially when the help is unsolicited, because of the freedom restriction involved in the help. Moreover, the norm of reciprocity in social exchange [12] suggests that, when receiving others’ help, individuals tend to generate feelings of inequity and indebtedness, a negative psychological state that refers to one’s strong desire and obligation to repay [8]. The norm of reciprocity perspective focuses on one’s immediate psychological reactions to help, so it is more appropriate than the other two theories are in predicting individuals’ state reactions to episodic help.

Resulting from feelings of inequity and indebtedness, employees receiving episodic help from colleagues are likely to experience ego depletion: “a temporary reduction in the self’s capacity or willingness to engage in volitional action” [19]. According to ego depletion theory, an individual’s ability to exert self-control is reduced when they engage in prior volitional acts that deplete their “ego resources” [19]. In other words, an individual is likely to deplete self-control resources (i.e., experience ego depletion) when they strive to inhibit thoughts or behaviors [31]. Drawing on these arguments, research showed that social interactions in the workplace create a resource-consuming context that often requires and depletes individuals’ self-control resources [32].

The norm of reciprocity is the central rule of a social exchange relationship between two parties [11]. Specifically, to maintain long-term-oriented, high-quality social exchange relationships with others, individuals are obligated to repay tangible and intangible resources received from others, leading to a positive, reciprocal relationship of exchanges between the two parties. In the organizational context, where colleagues have frequent social interactions and collaborations, interpersonal helping and receiving help are prevalent [4]. In such a context, when receiving colleagues for their help, employees have inner desires for reciprocity and feel indebted to repay [8,33]. Thus, before those help receivers can find a chance to repay the favor, they may use their self-control resources to regulate their thoughts of reciprocity and behavioral tendencies of repayment and compensation, leading to experiencing ego depletion. Partly in support of our argument, prior research showed that individuals tend to feel depleted when they seek to proactively impress others in the social-interaction process [32]. Thus, we propose the following:

**Hypothesis** **2** **(H2).** *Receiving help is positively related to the recipient’s ego depletion.*

### 2.3. Implications for Help Receivers’ Work Behaviors

Due to the ambivalent psychological states that help receivers generate, we now consider both positive and negative influences of receiving help on help receivers’ work behaviors via these two different psychological states. Previous studies showed that, when individuals are in a state of gratitude, they are generous to others [34] and appreciate their benefactor. Furthermore, gratitude is associated with prosocial tendencies and behavior due to its moral and positive valence [35]. On the other hand, when individuals are in a self-depletion state, they are less likely than their nondepleted peers are to engage in prosocial behavior [36], and they tend to exhibit deviant behaviors [37] and cheat behaviors [38]. Thus, through gratitude, employees tend to engage in more OCBs on the day when they receive help from colleagues. They may also engage in more deviant behaviors because of their experienced ego depletion.

Specifically, OCB is prosocial, and OCB performance is driven by both an individual’s prosocial motivation and the motivation to benefit the organization or members inside the organization [39]. Typical forms of OCB include helping newcomers to socialize within the organization and taking actions to protect the organization’s reputation and public impression [40]. Gratitude is conductive to one’s engagement of OCB for two main reasons. First, the norm of reciprocity suggests that feeling grateful and repaying kindness by giving one’s own resources to others or the organization is the balanced outcome of a social exchange relationship [8]. Indeed, empirical evidence showed that felt obligations and pressure to perform OCB are significant predictors of individual actual OCB performance [41]. Second, as a moral emotion, feeling gratitude tends to arouse one’s prosocial motivation to give and sacrifice [3,26]. For instance, prior research revealed that moral emotions of guilt, shame, and gratitude all contribute to more OCBs in the workplace [42]. Taken together, we propose:

**Hypothesis** **3** **(H3).** *Receiving help has a positive indirect relationship with the recipient’s OCB via gratitude.*

In contrast to OCB, deviance is employees’ intentional behaviors and acts that intend to cause harm to the organization or members inside the organization [20]. Typical forms of deviance include being late for work and engaging in verbal aggression [43]. Because such behaviors can satisfy employees’ current needs for psychological relief [44], self-control resources are crucial for preventing employees from performing deviance. Indeed, abundant empirical findings showed that ego depletion can cause unethical individual behaviors [37,45]. More recent studies directly confirmed the positive relationship between ego depletion and employee deviance [46,47]. Taken together, we propose:

**Hypothesis** **4** **(H4).** *Receiving help has a positive indirect relationship with the recipient’s deviance via ego depletion.*

## 3. Method

### 3.1. Participants and Procedure

Data were collected from a large Chinese bank with several branches located in a city in central China. With the chief executive officer’s permission and support, we asked the HR manager of this bank to post an advertisement to all full-time service employees (e.g., bank tellers, relationship managers, and financial consultants) stating that a research team was seeking interested employees to participate in an academic survey of workplace behavior. The recruitment poster also stated the time requirement for this survey (i.e., participation of 10 consecutive working days) and indicated that each participant would receive CNY 100 (about USD 15) for their participation.

A total of 143 service employees of this bank participated in this diary survey. In each branch, participants were gathered in a large meeting room, learning the survey procedure instructed by the first author. Then, they were asked to complete the initial survey, collecting data on their demographic information. A week later, participants started the diary survey assessing their daily receiving help, gratitude, ego depletion, OCB, deviance, and positive and negative emotions.

Sixteen employees did not finish and return their daily surveys, so the final sample consisted of 127 employees, resulting in a response rate of 88.81%. Of the 127 participants, the average age was 24.25 (SD = 8.21), and 86.60% were female. The majority of them were well-educated (99.21% had a college education or higher). 

### 3.2. Measures

The questionnaires were originally developed in English. Brislin’s [48] translation and back-translation procedure was followed to ensure the accuracy of the Chinese version. Participants were instructed to indicate how they felt at work during that day using seven-point Likert scales (1 = strongly disagree and 7 = strongly agree).

Receiving help: Following Uy et al. [5], we measured employees’ receiving help at work during the day using three items from the scale developed by Spence et al. [42]. These three items were: “someone went out of their way to help me today,” “I received help from others today,” and “today, I owe my colleagues favors.” Cronbach’s alpha was 0.86.

Gratitude: Employees’ gratitude was measured using five items from Spence et al. [42]. Sample items were “I feel grateful,” “I feel a warm sense of appreciation,” and “I am happy to have been helped by others.” Cronbach’s alpha was 0.90.

Ego depletion: Employee’s daily ego depletion was measured using five items that were widely used in prior diary studies [47]. Sample items were “today, I feel drained,” “my mind feels unfocused right now,” and “right now, it would take a lot of effort for me to concentrate on something.” Cronbach’s alpha was 0.93.

Organizational-citizenship behavior: Employee’s daily organizational-citizenship behavior was measured with five items adapted from Lee and Allen’s [40] 16 item scale. These five items were selected because they were the most likely to vary on a daily basis, and they could capture both the interpersonal and organizational components of OCB. Specifically, participants indicated the extent to which they “expressed loyalty toward the organization,” “took action to protect the organization from potential problems,” “demonstrated concern about the image of the organization,” “willingly gave my time to help others who had work-related problems,” and “showed genuine concern and courtesy toward my coworkers.” This five-item version of the OCB scale was validated in prior research conducted in China [47]. Cronbach’s alpha was 0.88.

Deviance: Employee daily deviance was measured with eight items adapted from Bennett and Robinson’s [43] 13-item scale. These eight items were selected on the basis of (a) their likelihood of varying on a daily basis, (b) their potential relevance to the bank context, and (c) their ability to capture both the interpersonal and organizational components of deviance. Sample items were “today, I worked on a personal matter instead of work for my employer,” “today, I spent too much time fantasizing or daydreaming instead of working,” and “today, I made fun of someone at work.” Cronbach’s alpha was 0.93.

Control variables: Prior research suggested that employee daily performance of OCB and deviance is affected by one’s state of positive and negative affects [49,50], and that individual general affective states are related to one’s emotional state of gratitude [42] and ego depletion [51]. Thus, we controlled for employees’ daily emotional experiences (i.e., positive and negative affects) in all analyses. Specifically, individual general positive affect was measured using five items: “inspired,” “alert,” “excited,” “enthusiastic,” and “determined” (α = 0.75), and individual general negative affect was measured using five other items: “afraid,” “upset,” “scared,” “nervous,” and “distressed” (α = 0.86). All these 10 items were adapted from the PANAS [52]. In addition, following the recommendations for analyzing diary data [53] and in accordance with prior diary studies [54], we controlled for the previous day’s measures of gratitude and ego depletion (i.e., the mediator), and OCB and deviance (i.e., the dependent variables) on the within-individual level in all of our analyses. To ensure that our findings were not unduly affected by the inclusion of these control variables, we also reanalyzed our data without controlling for positive emotions, negative emotions, and the previous day’s measures. The pattern of findings remained consistent regardless of whether the control variables were included in our models. 

### 3.3. Analytic Approach

Given the nested structure and multiple dependent variables in our model, we used multilevel-path analytical modeling to test our hypotheses. Model estimation was conducted using Mplus 7.0 software [55]. To test the main effects and the mediation relationships (i.e., Hypotheses 1–4), we initially estimated a multilevel mediation model that specified the Level 1 random-slope effects of receiving help on gratitude and ego depletion, the Level 1 random slope effects of gratitude on OCB and deviance, and the Level 1 random slope effects of ego depletion on OCB and deviance. Following Tofighi, West, and MacKinnon [56], the covariances among random-slope effects were also estimated for the purpose of estimating Level 1 indirect effects. We also modeled the direct effects of receiving help on our outcome variables (i.e., OCB and deviance). In addition, positive affect, negative affect, and all previous-day measures of gratitude, ego depletion, OCB, and deviance were added in this model as control variables. To facilitate the interpretation of the findings, we grouped mean centered Level 1 predictors and control variables to obtain an unbiased estimate of the within-individual level relationship [57].

In further testing the indirect effect of receiving help on OCB through gratitude, and the indirect effect of receiving help on deviance through ego depletion (i.e., Hypotheses 3 and 4), Monte Carlo simulations were conducted with 20,000 replications to compute 95% confidence intervals. Preacher and Selig [58] recommended these procedures to capture the asymmetric nature of the sampling distribution of indirect effects in multilevel models.

## 4. Data Analysis and Results

### 4.1. Descriptive Statics and Correlations

Means, standard deviations, reliabilities, and correlations are presented in Table 1. Within-individual correlations are presented below the diagonal, and between-individual correlations are presented above the diagonal. On the between-individual level, within-individual constructs were aggregated to the individual’s average score over the 10-day reporting period.

### 4.2. Factor Analysis

Multilevel confirmatory-factor analysis (MCFA) was conducted to confirm the hypothesized five-factor structure of receiving help, gratitude, ego depletion, OCB, and deviance while accounting for the nested structure of the data. Before conducting MCFA, deviance was packed into two indices representing two distinct subdimensions (i.e., deviance toward individuals and deviance toward organization). 

The hypothesized five factor model showed a good fit to the data: *χ*2 (160) = 1033.31, *p* < 0.001, CFI = 0.98, RMSEA = 0.07, SRMR = 0.03. This five-factor model fit the data better than a four-factor model did, grouping the two dependent variables (i.e., OCB and deviance): *χ*2 (164) = 9256.00, *p* < 0.001, CFI = 0.81, RMSEA = 0.21, SRMR = 0.17; Δ*χ*2 = 2615.98, Δ*df* = 4, *p* < 0.001, and another four-factor model grouping the mediating variables (i.e., gratitude and ego depletion): *χ*2(164) = 14105.31, *p* < 0.001, CFI = 0.70, RMSEA = 0.26, SRMR = 0.30; Δ*χ*2 = 4148.33, Δ*df* = 4, *p* < 0.001. Overall, the results of the MCFA support the discriminant validity among our daily focal constructs.

### 4.3. Variance Partitioning

We conducted variance partition to examine whether there was sufficient within-individual variance for our Level 1 variables (i.e., receiving help, gratitude, ego depletion, OCB, and deviance). These results are presented in Table 2; the percentage of within-individual variance of Level 1 variables in the current study ranged from 24.71% to 50.31%, indicating that there was sufficient within-individual variance for our Level 1 variable scores.

### 4.4. Hypothesis Testing 

**Figure 2:**
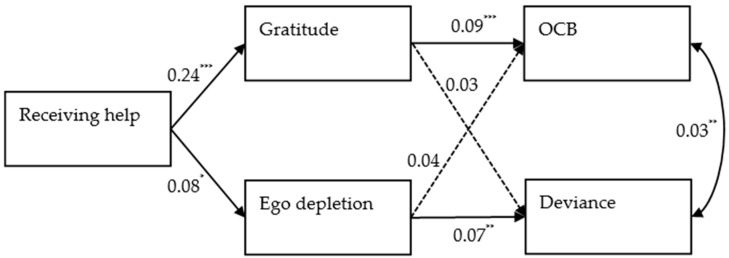
Unstandardized estimates of path coefficients. Note. OCB, organizational citizenship behavior. Solid lines, statistically significant paths; dashed lines, statistically nonsignificant paths. * *p* < 0.05, ** *p* < 0.01, *** *p* < 0.001.

In testing our research hypotheses, we conducted a multilevel mediation path analytical model, in which receiving help was included as the independent variable, gratitude and ego depletion were included as the mediators, and OCB and deviance were included as the dependent variables. Figure 2 presents parameter estimations for this path analytical model. Hypothesis 1 proposed the direct effect of receiving daily help on gratitude. The results, as shown in Table 3, revealed that receiving help daily was positively related to gratitude (*B* = 0.24, *p* < 0.001), supporting Hypothesis 1. Hypothesis 2 proposed the direct effect of daily receiving help on ego depletion. The results, as shown in Table 3, revealed that receiving help daily was positively related with ego depletion (*B* = 0.08, *p* < 0.05). Thus, Hypothesis 2 is supported.

Hypothesis 3 stated that gratitude mediated the effect of receiving daily help on OCB. Following Bauer, Preacher, and Gil [59], we computed the indirect effects as the product of *path a* (daily receiving help to gratitude) and *path b* (gratitude to OCB), plus the covariance between them (i.e., *indirect effect* = *path a* × *path b* + *cov* [a, b]). Bootstrapping results revealed that the indirect relationship between receiving help daily and OCB via gratitude was significant (*indirect effect* = 0.02, 95% *CI* = [0.009, 0.038]). Thus, Hypothesis 3 is supported. Hypothesis 4 stated that ego depletion mediated the effect of daily receiving help on deviance. Following the same procedures as those explained above, bootstrapping results revealed that the indirect relationship between receiving help daily and deviance via ego depletion was significant and positive (*indirect effect* = 0.01, 95% *CI* = [0.001, 0.014]). Thus, Hypothesis 4 is supported.

## 5. Discussion

Through a diary study using data collected from 127 full-time employees working in a large Chinese bank, we found a dual-path model of employees’ state psychological reactions to colleagues’ episodic helping behavior and the implications for their daily work behaviors. The findings of this study reveal ambivalent psychological states of help receivers in the context of receiving episodic help from others, that is, receiving help is positively related to both gratitude and ego depletion. Through these two psychological states, employees are likely to engage in OCB and deviance at work on the day when they receive help from colleagues. We next discuss theoretical and practical implications on the basis of these findings.

### 5.1. Theoretical and Practical Contributions

Although extensive studies explored individual reactions to help received from others or from society in the social-psychology literature, very few studies in the management literature focused on employees’ reactions to colleagues’ interpersonal help. Among these limited explorations, the dominant research findings suggest that receiving help from colleagues can benefit the help receiver to recover from work-related hindrance, pressure, and the loss of personal resources consumed at work. Our findings enrich this emerging stream of research by revealing that receiving episodic help from colleagues depletes employees’ self-control resources on a daily basis in the short run, in addition to making them feel grateful, as proposed by Fehr et al. [3]. These findings not only respond to the recent call for examining antecedents of employee gratitude in the organizational context [3,42], but also extend prior findings of individuals’ negative reactions to help in social-psychology research [6] by demonstrating ego depletion as a temporal, negative psychological state of help receivers.

Due to gratitude and ego depletion, employees receiving episodic help from colleagues behave more prosocially (i.e., OCB) and more destructively (i.e., deviance). These ambivalent findings contribute to the OCB literature in two aspects. Firstly, more recent OCB studies started to explore the negative outcomes of employees who previously engaged in OCB [24,42]. Instead of focusing on how OCB actors react, we shifted the research focus and examined how OCB targets (i.e., helping behavior) react both psychologically and behaviorally, providing an underexamined, target-centric perspective for future OCB research. 

Secondly, management scholars have devoted much attention to exploring the causal relationships between OCB and deviance, documenting empirical evidence supporting the effect that prior OCB leads to subsequent deviance, and the effect that prior deviance leads to subsequent OCB [60]. Our findings suggest that OCB and deviance, as two aspects of job performance, may coexist as ambivalent behavioral reactions motivated by employees’ complex psychological states in the workplace. 

Lastly, our research contributes to resource-related theories in the management literature. Specifically, previous research drawing on the theory of conservation of resources [61] suggests that receiving help can benefit individuals by lending them additional personal resources, such as time, money, and social support [9]. Drawing on the ego-depletion theory [20], our research focused on individuals’ self-control resources and revealed that receiving help from others is also a resource-consumption process in which help receivers consume self-control resources to regulate their inner desires for reciprocity and behavioral tendencies of repayment in the short run.

The findings from our study may be useful for managers as they try to create a positive climate that encourages mutual helping behaviors among workplace colleagues. First, managers should pay attention to the psychological reactions of employees who received help from others. Our findings show that help receivers may both feel grateful to others’ help, and also feel depleted in their self-regulatory processes due to the conflict between their inner desires for reciprocity and their current feelings of indebtedness. In addition, although employees who receive help are likely to reciprocate by engaging in more OCBs, managers should also anticipate that those who receive colleagues’ help may temporally engage in deviance at work due to their feelings of ego depletion.

Thus, to maintain a harmonious work environment of interpersonal helping, managers should try to remove or minimize help receivers depleted feelings associated with receiving help. One straightforward way is to decrease individuals’ indebtedness feelings by creating an organizational culture of companionate love. This culture should encourage high-quality relationships, increase coworker trust, and relieve the pressure of the obligation for reciprocity [62,63]. Moreover, managers can also create more opportunities for employees to participate into some prosocial activities (e.g., donation, public service, constructive voice), either inside or outside the organization. Through these prosocial activities, employees can repay the kindness received from others more easily and immediately, decreasing the period of ego-depletion feelings in the context of receiving help.

Furthermore, prior evidence shows that individuals who have a high construal level are more able to control their conduct, even when resources are depleted [64]. Along with high-level goals and values, a high construal level likely motivates individuals to overcome the negative effects of ego depletion [65]. Therefore, managers may select employees who have a high construal level in order to mitigate the negative consequences (i.e., deviance behaviors) of ego depletion for help receivers.

### 5.2. Limitations and Directions for Future Research

Although our study contributes to existing knowledge in several important aspects, it also has some limitations. One major limitation of our study is the utilization of cross-sectional designs. Although this cross-sectional design is quite common in prior diary studies [24,47], it prevents us from making causal conclusions on the basis of current empirical findings. Thus, it is possible that those who feel depleted because of work-related difficulties and problems are more likely to receive help from colleagues. The norm of reciprocity in social exchange [12] and prior social-psychology research on help receivers’ negative reactions [6] provide us with a strong theoretical basis for the effect that receiving help depletes one’s self-control resources.

Relatedly, we collected data from a single source, which is very likely to cause common method biases in our observed relationships. However, the satisfactory discriminant validity among the key research variables as shown in the CFA suggests that our data were not seriously affected due to common method variance. Moreover, consistent with prior diary studies [24], we controlled for previous-day measures on the focused dependent variables in data analyses to minimize the influences associated with common method biases and cross-sectional designs.

In addition to the aforementioned limitations related to research design, our findings cannot rule out alternative explanations as found in prior research. Specifically, although we controlled for help receivers’ general positive and negative emotions in demonstrating the mediating roles of gratitude and ego depletion linking receiving help with help receivers’ OCB and deviance, the negative, indirect relationship between receiving help and deviance could also be explained by the recipient’s inequity perception, decreased state self-esteem, and autonomy, as suggested in prior social-psychology research findings [6]. In a similar vein, the positive, indirect relationship between receiving help and OCB could be explained by other moral emotions, such as guilt and shame [60]. 

Lastly, one more limitation is the female over-representation in the sample. According to the social role theory [66], women are expected to be communal (e.g., friendly, unselfish, and emotionally expressive), while men are expected to be agentic (e.g., competent, assertive, and status-seeking). Gender differences in behavior are due to a mix of biologically and socially rooted factors, such as role expectations and internalized identities. Thus, in our sample, there may be some degree of bias in that women are more likely to feel gratitude and ego depletion after receiving help.

Given the above limitations, we offer several suggestions for future research. First, collecting experience-sampling data at multiple time points on every work day to create a time lag between independent and dependent variables may strengthen the robustness of our empirical findings. Such a design can also collect longitudinal data that can be used for testing the reciprocal processes between help giving and receiving. Second, although our findings revealed help receivers’ ambivalent psychological and behavioral reactions to others’ help, the conditions under which receiving help’s positive consequences can be magnified and negative consequences can be diminished remain unclear. Thus, we encourage future researchers to extend our findings by deeply examining both individual and contextual factors that can shape help receivers’ reactions to help. Lastly and more importantly, we suggest testing separate models for each gender in future research because of our sample bias. Such behaviors and attitudes are strongly related to gender-dependent personality traits and relationships between employees, since they may differ in terms of cause, process, and reaction in males and females.

## 6. Conclusions

Although accumulative empirical findings showed various consequences of OCB on actors themselves, very few studies examined OCB influences on target employees’ psychological and behavioral outcomes. Results from a diary study revealed that employees receiving occasional help at work generate ambivalent psychological states, with simultaneous enhanced feelings of gratitude and ego depletion. In turn, help receivers engage in both OCB and deviance at work on the day during which they receive others’ help. Overall, we made novel theoretical and empirical contributions to research that adopts a target-centric perspective in examining the consequences of OCB. Our research helps to facilitate researchers’ and practitioners’ understanding of and attempts to promote interpersonal help in the workplace.

## Figures and Tables

**Table 1:** Descriptive statistics and correlations among study variables.

Variables	Mean	SDw	SDb		Correlations					
				1	2	3	4	5	6	7
1. Positive affect	3.42	1.07	0.83	(0.75)	0.17	0.26 **	0.43 ***	−0.03	0.41 ***	0.03
2. Negative affect	2.42	1.07	0.85	0.15 ***	(0.86)	0.15	0.02	0.65 ***	−0.02	0.43 ***
3. Receiving help	4.31	1.37	1.04	0.17 ***	0.10 ***	(0.86)	0.65 **	0.14	0.56 ***	0.00
4. Gratitude	4.39	1.26	0.93	0.26 ***	0.04	0.47 ***	(0.90)	−0.13	0.64 ***	−0.05
5. Ego depletion	2.89	1.31	0.98	−0.06 *	0.49 ***	0.12 ***	−0.08 **	(0.93)	−0.18 *	0.62 ***
6. OCB	4.67	1.11	0.91	0.32 ***	0.02	0.46 ***	0.45 ***	–0.06 *	(0.88)	−0.27 **
7. Deviance	1.92	0.92	0.81	0.03	0.33 ***	−0.01	−0.02	0.45 ***	−0.17 ***	(0.93)

Note. Correlations above the diagonal represent between-individual correlations (*N* = 127). Correlations below the diagonal represent within-individual correlations (*N* = 1133). SDw (standard deviation in the within-person level) and SDb (standard deviation in the between-person level), standard deviations separately computed within and between individuals. Coefficient alpha estimates of reliability are in parentheses on the diagonal. OCB, organizational citizenship behavior. * *p* < 0.05; ** *p* < 0.01; *** *p* < 0.001.

**Table 2:** Percentage of within-individual variance among daily variables.

Daily Variables	Within-Individual Variance (e^2^)	Between-Individual Variance (r^2^)	Percentage of Within-Individual Variance
Receiving help	0.90	0.99	47.61%
Gratitude	0.80	0.79	50.31%
Ego depletion	0.86	0.86	50.00%
OCB	0.68	0.88	43.59%
Deviance	0.21	0.64	24.71%

Note: percentage of within-individual variance calculated as e^2^/ (e^2^ + r^2^).

**Table 3:** Multilevel path analysis results.

Variables	Gratitude		Ego Depletion		OCB		Deviance	
	Estimate	SE	Estimate	SE	Estimate	SE	Estimate	SE
Intercept	4.39 ***	0.09	2.84 ***	0.09	4.14 ***	0.18	1.53 ***	0.12
Control variables								
Positive affect	0.04	0.06	−0.16 **	0.05	0.12 ***	0.04	0.03	0.02
Negative affect	0.04	0.06	0.28 ***	0.05	0.05	0.03	0.03	0.03
Gratitude (t − 1)	0.01	0.04						
Ego depletion (t − 1)			0.16 ***	0.04				
OCB (t − 1)					0.15 **	0.06		
Deviance (t − 1)							0.10	0.07
Predictors								
Receiving help (t)	0.24 ***	0.04	0.08 *	0.04	0.18 ***	0.03	0.01	0.02
Gratitude (t)					0.09 ***	0.03		
Ego depletion (t)							0.07 **	0.03
Residual variance	0.68 ***	0.07	0.73 ***	0.07	0.36 ***	0.04	0.19 ***	0.03

Note. *N* = 127 at between-individual level; *N* = 1133 at within-individual level. “t,” evaluation “today”; “t − 1,” evaluation in previous day from “today.” The above estimates represent unstandardized path coefficients. All hypothesized effects calculated in the same path analytical model and specified as random slopes. * *p* < 0.05; ** *p* < 0.01; *** *p* < 0.001.

## Data Availability

Not applicable.
